# Anaplastic lymphoma kinase expression in *PDGFRA*-mutated gastrointestinal stromal tumors probably correlates with poor prognosis

**DOI:** 10.1186/s12957-023-03019-4

**Published:** 2023-04-29

**Authors:** Ying Wu, Beibei Gao, Qin Xia, Yili Zhu, Na Wang, Xiaona Chang, Bo Huang, Danju Luo, Jiwei Zhang, Peng Zhang, Heshui Shi, Jun Fan, Xiu Nie

**Affiliations:** 1 Department of Pathology, Union Hospital, Tongji Medical College, Huazhong University of Science and Technology, Wuhan 430022, Hubei, China; 2 Department of Gastrointestinal Surgery, Union Hospital, Tongji Medical College, Huazhong University of Science and Technology, Wuhan 430022, Hubei, China; 3 Department of Radiology, Union Hospital, Tongji Medical College, Huazhong University of Science and Technology, Wuhan 430022, Hubei, China

**Keywords:** Gastrointestinal stromal tumor, ALK expression, Clinicopathological features

## Abstract

**Background:**

Anaplastic lymphoma kinase (ALK) overexpression and gene alterations have been detected in several mesenchymal tumors, with significant implications for diagnosis, therapy and prognosis. However, few studies have investigated the correlation between ALK expression status and clinicopathological characteristics in patients with gastrointestinal stromal tumors (GISTs).

**Methods:**

A total of 506 GIST patients were enrolled. Sanger sequencing was employed to detect *c-KIT* and *PDGFRA* gene mutations. The tissue microarray (TMA) technique and immunohistochemistry were employed to identify the ALK (clone: 1A4 and D5F3) expression status in the tumor tissues. The ALK gene variants of IHC-positive cases were analyzed by fluorescence in situ hybridization (FISH) and next-generation sequencing (NGS). The clinicopathological data were analyzed using SPSS Statistics 26.0.

**Results:**

Among the 506 GIST patients, the *c-KIT* mutation accounted for 84.2% (426/506), followed by *PDGFRA* mutation (10.3%, 52/506), while the wild-type accounted for the least (5.5%, 28/506). ALK-positive expression was detected in *PDGFRA*-mutant GISTs (7.7%, 4/52) but negative for *c-KIT*-mutant or wild-type GISTs by IHC. Four ALK IHC-positive patients were all male. The tumors all occurred outside the stomach. The predominant patterns of growth were epithelioid (2/4), spindle (1/4), and mixed type (1/4). They were all identified as high-risk classification according to the National Institutes of Health (NIH) classification. Aberrant *ALK* mutations were not identified by DNA-based NGS except in one of the 4 cases with amplification by FISH.

**Conclusion:**

Our study revealed 7.7% (4/52) of ALK expression in *PDGFRA*-mutant GISTs, indicating that molecular tests were required to rule out the possibility of *PDGFRA*-mutant GISTs when encountering ALK-positive mesenchymal tumors with CD117-negative or weakly positive in immunohistochemical staining.

**Supplementary Information:**

The online version contains supplementary material available at 10.1186/s12957-023-03019-4.

## Introduction

Anaplastic lymphoma kinase (*ALK)* is a well-known driver oncogene in anaplastic large cell lymphoma (ALCL), non-small cell lung cancer (NSCLC) and renal cell carcinoma (RCC) and has been identified in other types of tumors, including inflammatory myofibroblast tumor (IMT), epithelioid inflammatory myofibroblast sarcoma (EIMS), and epithelioid fibrous histiocytoma (EFH). IMT is a rare neoplasm characterized by spindle-cell proliferation within an inflammatory infiltrate [1]. Approximately 50 to 60% of IMTs have activating rearrangements of the *ALK* gene on chromosome 2p23, resulting in constitutive tyrosine kinase activation, and crizotinib has proven to be an effective therapeutic approach. Recently, immunohistochemical expression of ALK in the majority of angiomatoid fibrous histiocytomas (AFHs) was demonstrated in an initial series by Cheah et al. and subsequently confirmed by Van Zwam et al. [2, 3]. Interestingly, the expression of ALK did not seem to be associated with *ALK* rearrangement or copy number variations. However, there are very few reports regarding the aberrant expression of ALK in gastrointestinal stromal tumors (GISTs).

GIST is the most common type of mesenchymal tumor and arises from Cajal cells in the gastrointestinal tract, with an annual incidence rate of 10–20 per million [4, 5]. Indeed, with the identification of activating mutations of either *c-KIT* or platelet-derived growth factor receptor (*PDGFRA*) tyrosine kinases as main players in GIST pathogenesis and development, in a few years, imatinib has become the backbone for the treatment of unresectable and advanced GISTs, the efficacy of which is profoundly affected by the underlying tumor genotype [6]. Tumors with the *PDGFRA D842V* mutation, which accounts for the majority of *PDGFRA*-mutant GISTs, are primary imatinib resistant but have been reported to be sensitive to avapritinib [7–10]. Recently, our group reported a case of *PDGFRA*-mutant GIST with ALK expression. According to the clinicopathological features and the National Institutes of Health (NIH) classification, the risk of recurrence was classified as high [11]. Herein, we aimed to investigate the association between the expression of ALK and the clinicopathological characteristics of GISTs.

## Materials and methods

### Patients and tumor samples

We enrolled a total of 506 patients who had a confirmatory diagnosis of GIST after surgical resection or biopsy and were all sequenced by the Sanger sequencing method in the Union Hospital, Tongji Medical College, Huazhong University of Science and Technology, from January 2015 to June 2022. The diagnoses were all confirmed through histopathological, immunohistochemical, and molecular tests. Hematoxylin and eosin (HE)-stained slides and formalin-fixed paraffin-embedded (FFPE) tissue blocks were obtained from the Department of Pathology. The study was approved by the Ethics Committee of Union Hospital (Ethical Approval Number: S-377). All patients signed informed consent forms before the initiation of the study-related procedure.

### Tissue microarray

The tissue microarray (TMA) technique was performed as described previously [12, 13]. HE slides were screened to identify optimal tumor tissue for analysis. A red pen was used to mark representative areas on the ‘donor’ FFPE GIST blocks. TMA blocks were then constructed (DAN JIER Electronics Co., Ltd, JiNan, China). Three core tissue biopsies (diameter, 1.0 mm) were collected from non-necrotic areas of tumor foci in each individual ‘donor’ FFPE GIST block and precisely arrayed into a new ‘recipient’ paraffin-embedded block (24 × 37 mm) using a custom-built instrument. The TMA was placed on top of a glass slide and heated to 55 °C for 15 min to securely bind and level the cores to the ‘recipient’ block. The glass slide and TMA block ensemble were then transferred to an ice pack for 30 min to complete construction. Sections (4 μm) of the ‘recipient’ TMA blocks were made using standard techniques. Due to the biopsy GIST samples lacking sufficient tumor tissues, the TMA technique was performed only in surgical GIST samples. The biopsy GIST FFPE tissue blocks were cut into 4 μm sections for immunohistochemistry staining.

### Immunohistochemistry

Paraffin Sects. (4 μm) for immunohistochemistry were cut from ‘recipient’ TMA blocks and biopsy GIST FFPE tissue blocks. Immunohistochemical staining was performed by the Envision two-step method. The expression of ALK tyrosine kinase was examined using two monoclonal antibodies against ALK (Ventana Medical System, Inc.; Clone: 1A4 and D5F3). The other immunohistochemical indicators referred to archived sections, including CD117 (Ventana Medical System, Inc., Tucson, AZ, USA; Clone: c-kit), DOG1 (MXB, Fuzhou, China; Clone: SP31), CD34 (Gene Tech, Shanghai, China; Clone: QBEnd 10), desmin, smooth muscle actin (SMA) (Gene Tech; Clone: 1A4), SDHB (ZSGB-BIO, Beijing, China; Clone: OTI1H6), S-100 protein (MXB, Fuzhou, China; Clone: 4C4.9), and Ki-67 (MXB, Clone: MIB-5).

### Fluorescence in‐situ hybridization

Fluorescence in situ hybridization (FISH) was performed on 4‐μm‐thick FFPE sections obtained from ALK IHC-positive patients using the Vysis dual-color *ALK* break-apart probe kit (Abbott Molecular/Vysis, Des Plaines, IL, USA) following the manufacturer’s instructions. The *ALK* gene copy number was counted in a minimum of 50 nonoverlapping nuclei in select cases.

### Sanger sequencing

The Sanger sequencing method was used to detect *c-KIT (*exon 9, 11, 13, and 17*)* and *PDGFRA* (exon 12 and 18*)* gene mutations in all cases. According to the kit (*c-KIT* and *PDGFRA* gene mutation detection kit of Xiamen Aide Co., Ltd, Xiamen, China) instructions, genomic DNA was extracted from the samples. *C-KIT* exons 9, 11, 13, and 17 and *PDGFRA* exons 12 and 18 were amplified with polymerase chain reaction (PCR). After detecting the PCR products by gel electrophoresis, the PCR products were sequenced using ABI 3730XL DNA sequencers. The sequencing results were compared with the standard template sequences of the BLAST program within the CHROMAS software to identify the gene mutation loci.

### Next-generation sequencing

FFPE tissue blocks for next-generation sequencing (NGS) were obtained from ALK IHC-positive patients. NGS was performed by Tongshu Gene (Shanghai, China). The OncoPanel consists of targeted sequencing of 556 cancer-related genes. The 23 fusion genes in the 556 panel were detected by DNA sequencing, including *ALK* rearrangement, amplifications or activation mutations.

### Reverse transcription-polymerase chain reaction

For comparison between the tumor and normal tissues, ALK expression in tumor tissues was calculated using normal tissue sample expression as a reference control. Only in cases 1 and 3, tumor tissues and normal control tissues were available. FFPE tissue blocks for reverse transcription-polymerase chain reaction (RT–PCR) were obtained from surgically resected specimens in cases 1 and 3. For each case, tumor tissues and adjacent normal tissues were randomly selected in this experiment. Total RNA was extracted from paraffin-embedded tissue sections and isolated by an RNA extraction kit (AmoyDx, Xiamen, China) according to the manufacturer's instructions. Then, cDNA was obtained via reverse transcription (PrimeScriptTM II 1st Strand cDNA Synthesis Kit, TaKaRa, Dalian, China). PCR was performed using 2 × Hieff ® Robust PCR Master Mix (With Dye) (Yeasen, Shanghai, China). The primers included human ALK, forward 5′- AACCACTTCATCCACCGAGACATTG -3′ and reverse 5′- TGTTGCCAGCACTGAGTCATTATCC-3′. Human glyceraldehyde-3-phosphate dehydrogenase (GAPDH) was used as an endogenous reference gene primer to normalize human ALK expression. The expected lengths of the PCR fragments were 377 and 138 bp. The cDNA (5 µl/lane) was separated by 40% polyacrylamide gel electrophoresis (PAGE). Five microliters of DNA marker liquid (code no. 3590Q; TaKaRa, Dalian, China) was used to separate PCR products, the results of which were observed using a gel imaging analyzer (Tanon, Shanghai, China). Due to the lack of adjacent normal tissues as a reference, the patients in cases 2 and 4 did not undergo RT–PCR.

### Follow-up and statistical analysis

The 52 *PDGFRA-*mutant patients were followed up by telephone and the outpatient service at regular intervals (3- to 6-month intervals postoperatively for 2 years and annually thereafter). Overall survival (OS) was defined as the time from diagnosis to death. Statistical analysis was conducted in SPSS 26.0 for Windows (SPSS, Chicago, IL, USA). The median or mean ± SD was used to express the measurement data, and overall survival curves were drawn by the Kaplan–Meier (K-M) method.

## Results

### Types of gene mutations

**Fig. 1:**
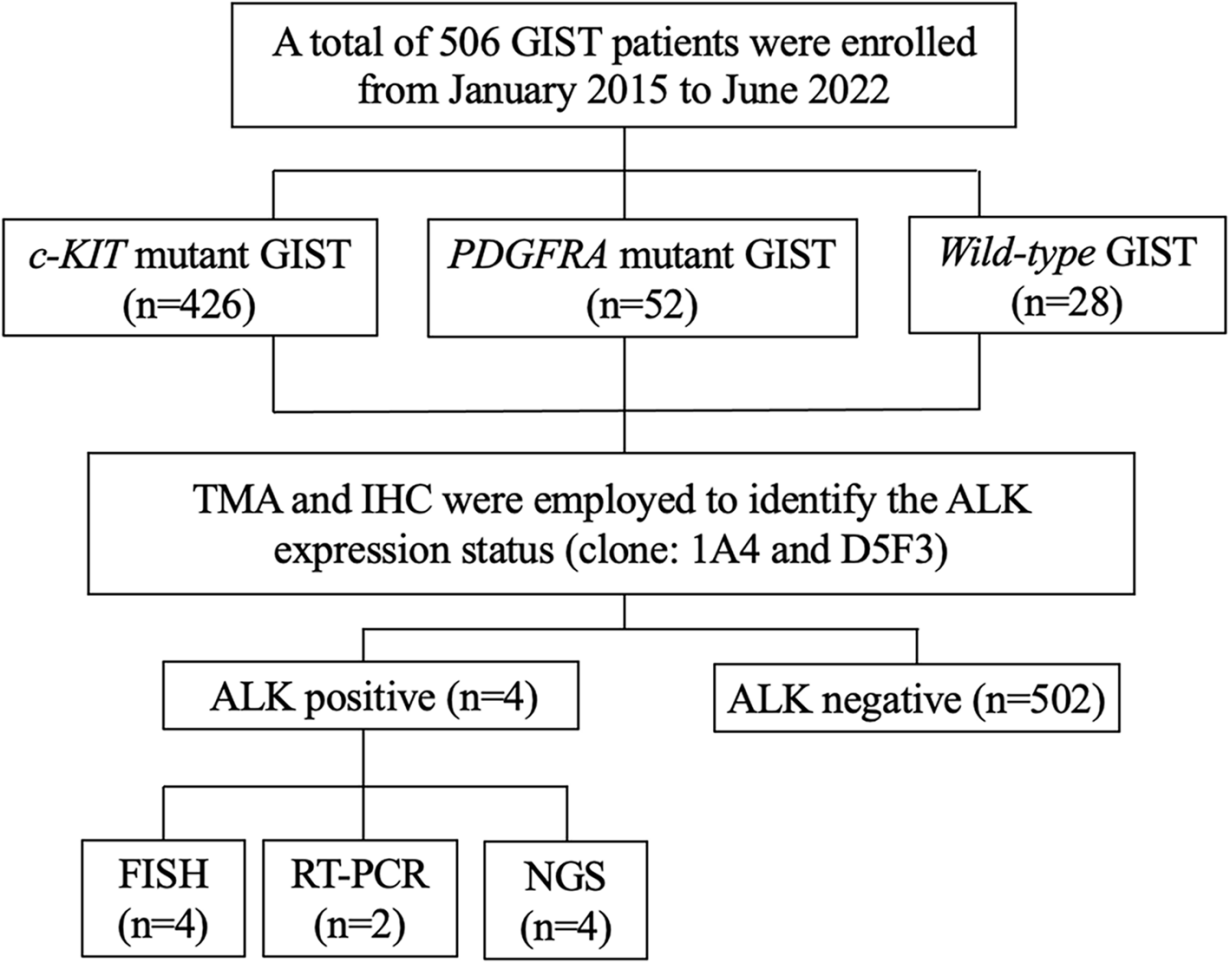
Flow diagram of the study design

Figure 1 summarizes our study design. A total of 506 GIST patients were enrolled in our study. After sequenced by Sanger sequencing method, the mutation types were identified: 426 patients with *c-KIT* mutation, 52 patients with *PDGFRA* mutation and 28 patients with wild-type mutation. Employed with TMA and IHC, four ALK-positive cases and 502 ALK-negative cases were revealed by TMA and IHC. To explore the molecular basis of ALK expression, FISH and NGS were performed in four ALK-positive cases. Among them, two were conducted with RT‒PCR.

Among the 506 GIST patients, the *c-KIT* mutation was predominant (426, 84.2%), followed by the *PDGFRA* mutation (52, 10.3%), while the wild-type was the least (28, 5.5%). Deletion and point mutation were the most frequent mutational patterns (30.2%, 153/506 for both), followed by mixed (16.2%, 82/506) and duplication (13.4%, 68/506). Insertion was relatively fewer, accounting for 2.0% (10/506). In the *c-KIT* mutation, deletion in exon 11 occurred most frequently (29.6%, 150/506), while in the *PDGFRA* mutation, the most frequent type was mutations in exon 18 (9.1%, 46/506), followed by mutations in exon 12 (1.2%, 6/506). The detailed mutation types of all patients are summarized in Supplementary Table S1.

### ALK protein expression and gene variant status

ALK (1A4 and D5F3) immunohistochemical stains were used in 506 GIST cases, of which 4 cases showed strong positivity. The positive cases were all identified in *PDGFRA* mutation (7.7%, 4/52), while none in the *c-KIT* mutation or wild-type mutation. No *ALK* rearrangement, amplifications or activation mutations were identified for the 4 ALK IHC-positive cases according to the DNA-based sequencing, but one was identified as amplification by FISH.

### Clinicopathological features of 4 ALK-positive patients

The 4 ALK IHC-positive patients were all male. The patients’ median age was 55.5 years old (range from 37 to 66 years old). Among them, 3 (75%, 3/4) patients were younger than 60 years old. The tumors of 4 ALK-positive patients all occurred outside the stomach. The maximum sizes of the tumors were all greater than 10 cm, and the mitotic accounts of 3 ALK-positive patients (75%, 3/4) were more than 10/5 mm^2^. The predominant patterns of growth were epithelioid (2/4), spindle and mix type (1/4). They were all identified as high-risk classification according to NIH criteria, 2008 modified version. A review of the clinicopathological features and prognosis of 4 ALK-positive patients is provided in Table 1. And the demographic and clinicopathological features of ALK-positive group and ALK negative group were summarized in Supplementary Table S2.

**Table 1:** The clinicopathological features and prognosis of 4 ALK-positive patients

Case no	1	2	3	4
Sex	Male	Male	Male	Male
Age (years old)	37	57	66	53
Primary site	Transverse colon	Left abdomen	Right middle abdomen	Ileum
Time of diagnosis	June 20, 2018	September 25, 2019	December 29, 2020	March 13, 2017
Histological type	Epithelioid	Epithelioid	Epithelioid and spindle	Spindle
Maximum size of tumor (cm)	18	18.5	15.7	12
Mitotic accounts (/5 mm^2^)	> 5	> 10	> 10	> 10
Recurrence risk	High	High	High	High
IHC				
ALK(1A4)	( +)	( +)	( +)	( +)
ALK(D5F3)	( +)	( +)	( +)	( +)
PDGFRA mutation type	Exon 18 D842V	Exon18 D842 _D846delinsE	Exon 18 D842V	Exon 12 D583G
ALK FISH				
Translocation	( −)	( −)	( −)	( −)
Amplification	( −)	( −)	( +)	( −)
NGS ALK				
Fusion	( −)	( −)	( −)	( −)
CNI	( −)	( −)	( −)	( −)
Recurrence time	February 2019	NA	NA	July 16, 2018
Recurrence site	liver	NA	NA	Pelvic cavity
Treatment after recurrence	Dasatinib, PD,1mth Ceritinib, PD, 1 month	NA	NA	Surgery
OS (month)	25 (dead)	2 (dead)	2 (alive)	49 (dead)

*NA* not available, *PD* progressive disease, *CNI* copy number increase

### CT imaging, morphology, IHC, and FISH of the 4 ALK-positive patients

**Fig. 2:**
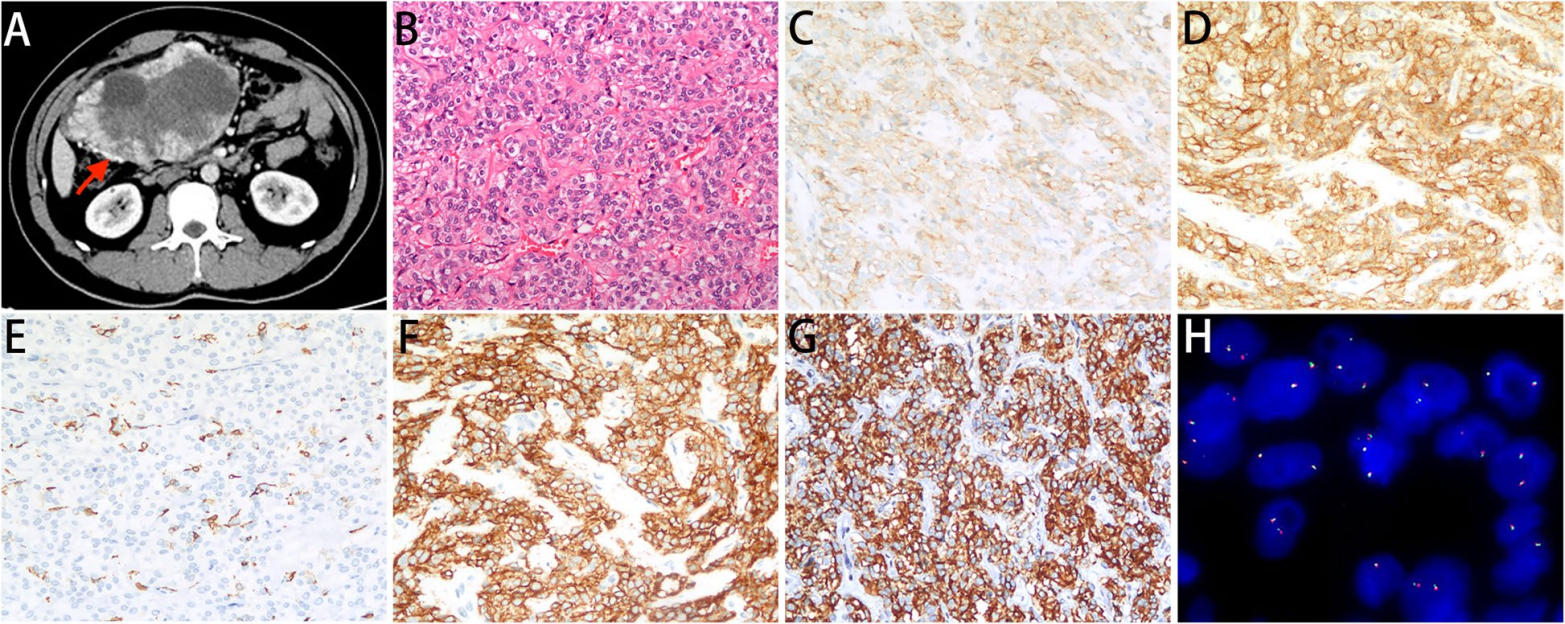
The examination images of case 1. **A** Abdominal CT scan shows an uneven density shadow sized 16.2 × 15.4 × 8.8 cm on the right upper abdominal cavity (red arrow). **B** The tumor was consisted of epithelioid tumor cells by H&E staining (200 ×). **C**–**E** (200 ×) The tumor cells showed a positive signal for CD117 (**C**) and DOG1 (**D**) and a negative signal for SMA (**E**). **F**–**G** ALK IHC showed strong positive staining for 1A4 (**F**) and D5F3 (**G**). **K** FISH did not reveal an *ALK* rearrangement (1000 ×)

The examination images of case 1 are shown in Fig. 2. Computed tomography (CT) revealed an uneven density shadow sized 16.2 × 15.4 × 8.8 cm on the right upper abdominal cavity (Fig. 2A). Postoperative pathological examination revealed a large number of epithelioid tumor cells (Fig. 2B) with regional cystic degeneration, hemorrhage and necrosis. Immunohistochemical staining showed positive expression of CD117 (weakly) and DOG-1 and negative expression of SMA (Fig. 2C, D, E). ALK expression was strong in tumor cells with two different antibody clones, 1A4 (Fig. 2F) and D5F3 (Fig. 2G). Fluorescent in situ hybridization (FISH) using a break-apart probe showed no *ALK* rearrangement (Fig. 2H). Information on cases 2–4 was shown in the corresponding supplementary material (Supplementary Figures S1–S3).

### Relative ALK mRNA expression

The electrophoresis results of case 1 and case 3 were shown in Supplementary Figure S4. Electrophoresis analysis showed uniform density between the band in lane 3 and the band in lane 4, which lay in the < 150-bp area and were consistent with the theoretical amplification length (138 bp). In Lane 1, no band was observed. Compared with lane 1, a faint band at ≈ 400 bp (theoretical amplification length 377 bp) was observed in lane 2. The bands in lanes 7 and 8 also exhibited a uniform density, located at ≈ 150 bp. In lane 5, no association of the ALK band was detected. In lane 6, a faint band appeared at ≈ 400 bp (theoretical amplification length 377 bp).

### Survival of 52 PDGFRA-mutant patients

**Fig. 3:**
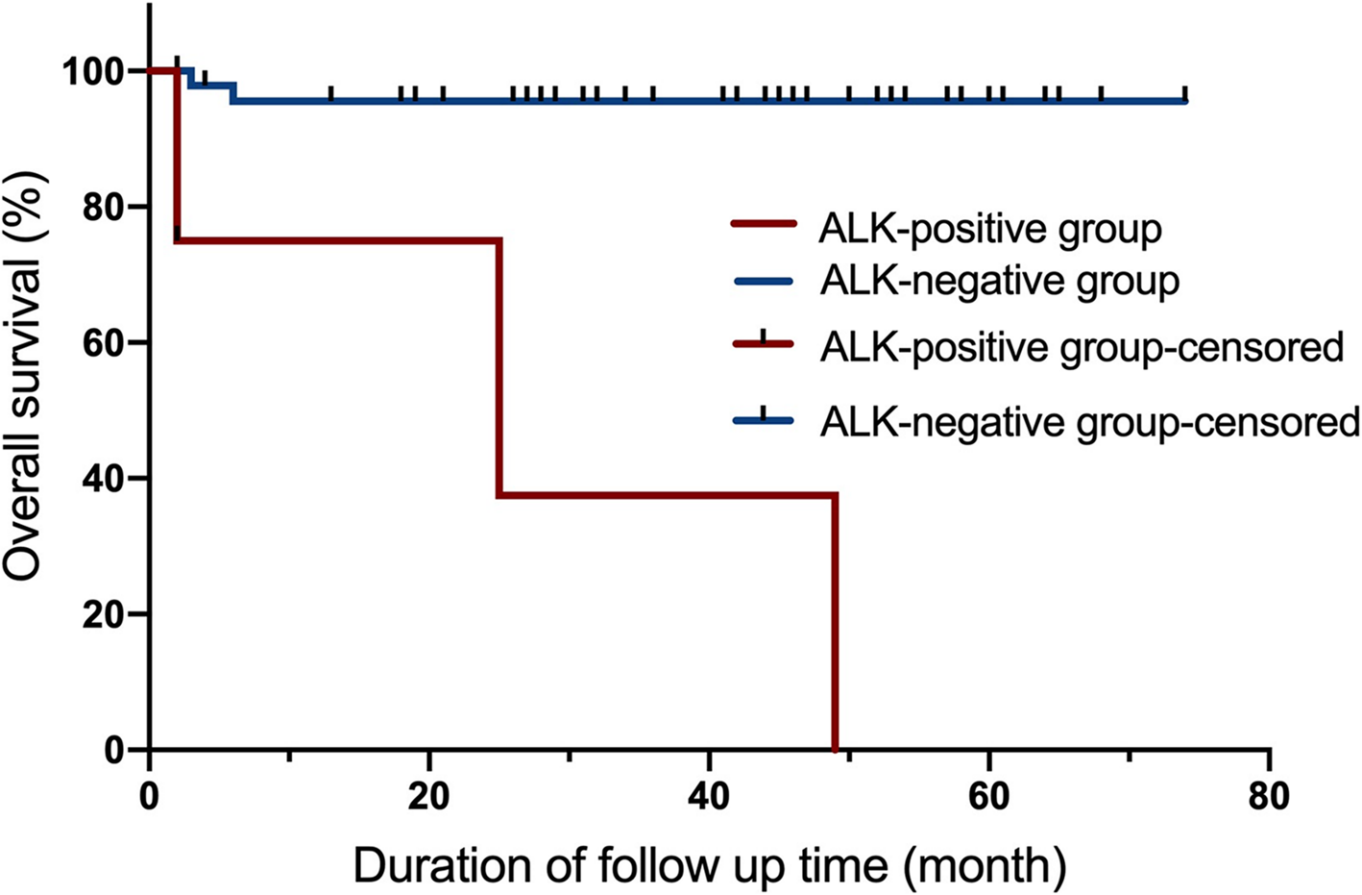
The K-M plot for overall survival of ALK-positive group and ALK-negative group

The 52 patients were followed until death or loss to follow-up (F/U). The median OS of the ALK-positive group (*n* = 4) and ALK-negative group (*n* = 48) was 25.00 months and not reached, respectively. Three ALK-positive patients (75%) died at 2, 25, and 49 months after diagnosis, and 2 ALK-negative patients (3.6%) died at 3 and 6 months after diagnosis. The OS was shown in K-M curves (Fig. 3).

## Discussion

*ALK*, a transmembrane tyrosine kinase, belongs to the superfamily of insulin receptors that regulate cellular growth and may trigger neoplastic transformation, including platelet-derived growth factor receptors, epidermal growth factor receptor, human epidermal growth factor receptor type 2, and insulin-like growth factor-1 receptors [14]. Aberrantly formed *ALK* oncogenes, mainly caused by fusion mutations, *ALK* gain-of-function mutations, or *ALK* amplification, have been identified in various cancers. However, the aberrant expression of ALK in GIST has rarely been reported. Recently, our group reported a case of *PDGFRA*-mutant GIST with ALK expression, in which the tumor had an immense size, and follow-up revealed liver metastases that developed quickly [11]. In this study, a total of 506 GIST patients were included. ALK-positive expression was not identified in *c-KIT*-mutant or wild-type GISTs, while the rate of ALK expression among *PDGFRA-*mutant GISTs was 7.7% (4/52) without aberrant *ALK* mutations. Furthermore, the ALK-positive expression cases exhibited more aggressive biological behavior and a worse overall prognosis.

*PDGFRA-*mutant GISTs preferentially occur in the stomach, occasionally outside the stomach, including the intestine, abdominal cavity, and mesentery [15]. The histological types of *PDGFRA-*mutant GISTs are mainly epithelioid or mixed subtype and are rarely spindle cell type [15]. Immunostaining often shows poor expression of CD117 and DOG1 [16]. ALK expression in GISTs with *PDGFRA* mutations can potentially lead to difficulty in differentiating them from IMTs, more than 50% of which displayed *ALK* gene rearrangement and overexpression. Here, we reported that 7.7% of GISTs with *PDGFRA* mutations showed strong and diffuse staining for ALK, which was confirmed by antibodies from two different clones in our research. Therefore, GISTs should also be taken into account in the differential diagnosis of ALK-positive soft tissue tumors, especially IMT, because the suggested treatment regimens for IMT and GIST are strikingly different. Therefore, genetic testing is crucial for differential diagnosis.

In general, studies focused on *ALK* gene alterations and expression in GISTs are rare. Zhao Lei et al. reported a GIST occurred in the small bowel with the ALK protein overexpression. The NGS sequencing identified a rearrangement involving *PPP1R21* and *ALK*. No relapse was observed at the 8-month follow-up for the patient [17]. Recently, Huang et al. [18] reported a quadruple wild-type (qWT) GIST with ALK (D5F3) overexpression, and a novel *CDC42BPB-ALK* fusion was identified by NGS. However, the two cases both lacked known GIST driver genes, such as *KIT*, *PDGFRA*, *BRAF*, and the *SDHx* gene family. Thus, we think it is difficult to rule out the possibility of *ALK*-rearranged mesenchymal tumors with the Cajal cell phenotype and positive expression of CD117 and DOG-1.

To the best of our knowledge, there are few reports about ALK protein expression without *ALK* gene abnormalities in soft tissue tumors. Similarly, John R. Goldblum and colleagues reported a high frequency (9/11) of ALK positivity in AFH [17], which is an *EWSR1* (or rarely *FUS*) rearrangement-associated neoplasm characterized by accompanying lymphoplasmacytic infiltrate. However, the molecular basis for ALK immunoreactivity in AFH in their series is unclear. In our study, two available cases of pairwise comparison RT–PCR revealed increased *ALK* mRNA expression in tumors compared with adjacent normal tissues but without *ALK* gene rearrangement, amplifications or activation mutations detected by DNA-based NGS. Therefore, we speculated that *PDGFRA* mutation was probably involved in upregulating the expression of the *ALK* gene, and the underlying molecular mechanisms remain to be explored.

GISTs with *PDGFRA* mutations are believed to involve an indolent process and exhibit a favorable prognosis [19, 20]. However, our team recently reported the first case of ALK expression in a *PDGFRA*-mutated GIST, and follow-up revealed liver metastases that developed quickly [11]. According to our limited data, GIST patients with both ALK expression and *PDGFRA* mutation tend to have a larger tumor diameter, higher recurrence risk and more mitotic figures, indicating that ALK expression may be associated with a worse prognosis. Our follow-up results were broadly consistent with the above speculation. Three of 4 ALK-positive patients presented progression within the first 2 years, and one of these patients with *PDGFRA D842V* mutation experienced liver metastasis only 6 months after surgical treatment. In general, ALK-positive patients exhibited invasive biological behavior and a worse prognosis. The patient in case 1 responded poorly to the second-generation *ALK*-TKI ceretinib after treatment with first-line dasatinib. We presumed that ALK expression might be caused by epigenetic modification or other mechanisms rather than the main driver gene, which results in disease progression.

Indeed, our study has several limitations, including the retrospective, single-center design. To our knowledge, it was probably inappropriate to statistically compare the only 4 patients with ALK-positive disease with the remaining PDGFRA mutant cases, which may result in some error in the reliability and accuracy of the statistical results for survival analysis. In addition, the mechanism of ALK expression and the unfavorable outcome in *PDGFRA* mutation patients is unclear.

In summary, our study explored the ALK expression status in GISTs and revealed ALK overexpression in 7.7% *PDGFRA*-mutant GISTs specifically. The results indicated that *PDGFRA*-mutant GISTs should be considered a differential diagnosis when encountering an ALK-positive mesenchymal tumor, especially those with CD117-negative or weakly positive in immunohistochemical staining. If necessary, molecular detection should be carried out for further clarification. In addition, the expression of ALK may be a factor leading to more invasive biological behavior and poor prognosis in ALK-positive PDGFRA-mutated GISTs.

## Supplementary Information

**Additional file 1: Supplementary Table S1.** Mutation types of 506 patients by Sanger sequencing.

**Additional file 2: Supplementary Table S2.** Demographic and clinicopathological features of ALK-positive group and ALK-negative group.

**Additional file 3: Supplementary Figure S1.** Examination images of case 2. (A) CT revealed an irregular mass sized 18.5×7.3×28.2 cm located in the left abdominal cavity. The margins of the lesion could not be clearly discriminated from the stomach and intestinal walls (red arrow). (B) The tumor showed an epithelioid growth pattern by H&E staining (200×). (C) The tumor cells showed a positive cytoplasmic signal for CD117 (200×). (D) The tumor cells showed a positive cytoplasmic signal for DOG1 (200×). (E) The tumor cells showed a negative cytoplasmic signal for SMA (200×). (F) ALK IHC showed positive strong and diffuse staining by the 1A4 clone (200×). (G) ALK IHC showed positive strong and diffuse staining by the D5F3 clone (200×). (H) A break-apart fluorescent in situ hybridization (FISH) assay did not reveal *ALK* rearrangement (1000×). **Supplementary Figure 2.** Examination images of case 3. (A) Preoperative CT showed a mixed slightly low-density mass of approximately 15.7×7.6 cm in the right-middle abdomen. Contrast-enhanced scanning showed uneven enhancement, with multiple areas of unenhanced necrosis (red arrow). (B) The tumor demonstrated epithelioid and spindle growth by H&E staining (200×). (C) The tumor cells showed a positive cytoplasmic signal for CD117 (200×). (D) The tumor cells showed a positive cytoplasmic signal for DOG1 (200×). (E) The tumor cells showed a negative cytoplasmic signal for SMA (200×). (F) ALK IHC showed strong positive staining by the 1A4 clone (200×). (G) ALK IHC showed strong positive staining by the D5F3 clone (200×). (H) A break-apart fluorescent in situ hybridization (FISH) assay did not identify ALK rearrangement, but some tumor cells were identified as having a copy number >2 (1000×). **Supplementary Figure 3.** The examination images of case 4. (A) Examination CT showed uneven enhanced density shadows in the pelvic cavity, right rectus abdominus region and deep right abdominal wall, the maximum size of which was 11.0 cm×6.7 cm (red arrow). (B) The tumor demonstrated a spindle growth pattern stained by H&E staining (200×). (C) The tumor cells showed a positive cytoplasmic signal for CD117 (200×). (D) The tumor cells showed a positive cytoplasmic signal for DOG1 (200×). (E) The tumor cells showed a positive cytoplasmic signal for SMA (200×). (F) ALK IHC showed positive staining by the 1A4 clone (200×). (G) ALK IHC showed strong positive staining by the D5F3 clone (200×). (H) A break-apart fluorescent in situ hybridization (FISH) assay did not reveal *ALK* rearrangement (1000×). **Supplementary Figure 4.** PAGE electrophoresis results of RT-PCR products of two available pairwise tissues. Lane 1-4 and 5-8 show the electrophoresis results of case 1 and case 3. Lane M, DNA marker; Lane 1/5, PCR amplification product obtained with *ALK *gene primers from normal tissues; Lane 2/6, PCR amplification product obtained with *ALK* gene primers from tumor tissues; Lane 3/7, PCR amplification product obtained with *GAPDH* gene primers from normal tissues; Lane 4/8, PCR amplification product obtained with *GAPDH* gene primers from tumor tissues.

## Data Availability

The datasets used and/or analyzed during the current study are available from the corresponding author on reasonable request.

## Abbreviations

ALK: Anaplastic lymphoma kinase
ALCL: Anaplastic large cell lymphoma
NSCLC: Non-small cell lung cancer
RCC: Renal cell carcinoma
IMT: Inflammatory myofibroblast tumor
EIMS: Epithelioid inflammatory myofibroblast sarcoma
EFH: Epithelioid fibrous histiocytoma
AFHs: Angiomatoid fibrous histiocytomas
GIST: Gastrointestinal stromal tumor
PDGFRA: Platelet-derived growth factor receptor
NIH: National Institutes of Health
HE: Hematoxylin and eosin
FFPE: Formalin-fixed paraffin-embedded
TMA: Tissue microarray
FISH: Fluorescence in situ hybridization
NGS: Next-generation sequencing
RT–PCR: Reverse transcription-polymerase chain reaction
GAPDH: Glyceraldehyde-3-phosphate dehydrogenase
PAGE: Polyacrylamide gel electrophoresis
OS: Overall survival
K-M: Kaplan–Meier
CT: Computed tomography
qWT: Quadruple wild-type
