# Supplementary material for: Anaplastic lymphoma kinase expression in PDGFRA-mutated gastrointestinal stromal tumors probably correlates with poor prognosis
Source: World J Surg Oncol. 2023 Apr 29;21:138. doi: 10.1186/s12957-023-03019-4 (PMC10148552; doi:10.1186/s12957-023-03019-4)
Supplement: Supplementary file 1 — Additional file 1: Supplementary Table S1. Mutation types of 506 patients by Sanger sequencing. [file 12957_2023_3019_MOESM1_ESM.docx]

**Supplementary Table 1.** Mutation types of 506 patients by Sanger sequencing

| Gene Alteration | Number  (%) | Mutation types | | | | |
| --- | --- | --- | --- | --- | --- | --- |
|  |  | Deletion  N(%) | Point  N(%) | Insertion N(%) | Duplication  N(%) | *Mixed N(%) |
| Wild type | 28(5.5) | 0 | 0 | 0 | 0 | 0 |
| Mutation | 478(94.5) | 153(30.2) | 153(30.2) | 10(2.0) | 68(13.4) | 82(16.2) |
| c-KIT | 426(84.2) | 150(29.6) | 113(22.3) | 10(2.0) | 68(13.4) | 73(14.4) |
| Exon 9 | 47(9.3) | 0 | 1(0.2) | 0 | 46(9.1) | 0 |
| Exon 11 | 363(71.3) | 150(29.6) | 108(21.3) | 10(2.0) | 22(4.3) | 73(14.4) |
| Exon 13 | 13(2.6) | 0 | 2(0.4) | 0 | 0 | 0 |
| Exon 17 | 3(0.6) | 0 | 2(0.4) | 0 | 0 | 0 |
| PDGFRA | 52(10.3) | 3(0.6) | 40(8.0) | 0 | 0 | 9(1.8) |
| Exon 12 | 6(1.2) | 1(0.2) | 1(0.2) | 0 | 0 | 4(0.8) |
| Exon 18 | 46(9.1) | 2(0.4) | 39(7.7) | 0 | 0 | 5(1.0) |

*Mixed=Insertion+Deletion
