# Supplementary material for: Anaplastic lymphoma kinase expression in PDGFRA-mutated gastrointestinal stromal tumors probably correlates with poor prognosis
Source: World J Surg Oncol. 2023 Apr 29;21:138. doi: 10.1186/s12957-023-03019-4 (PMC10148552; doi:10.1186/s12957-023-03019-4)
Supplement: Supplementary file 2 — Additional file 2: Supplementary Table S2. Demographic and clinicopathological features of ALK-positive group and ALK-negative group. [file 12957_2023_3019_MOESM2_ESM.docx]

| **Supplementary Table 2.** Demographic and clinicopathological features of ALK-positive group and ALK-negative group | | |
| --- | --- | --- |
| Clinicopathologic Feature | *ALK* positive(n=4) | *ALK* negative(n=48) |
| Sex-No.(%) | | |
| Male | 4 (100.0) | 36 (75.0) |
| Female | 0 (0.0) | 12 (25.0) |
| Age of onset-No.(%) | | |
| ≥60 | 1 (25.0) | 17 (35.4) |
| ＜60 | 3 (75.0) | 31 (64.6) |
| Primary site-No.(%) | | |
| Stomach | 0 (0.0) | 39 (81.2) |
| Outside the stomach | 4 (100.0) | 9 (18.8) |
| Maximum tumor diameter(cm) -No.(%) | | |
| ≤5 | 0 (0.0) | 20 (41.7) |
| 5-10 | 0 (0.0) | 18 (37.5) |
| ＞10 | 4 (100.0) | 10 (20.8) |
| Histocyte morphology-No.(%) | | |
| spindle cell type | 2 (50.0) | 16 (33.3) |
| epithelioid cell type | 1 (25.0) | 21 (43.8) |
| mixed subtype | 1 (25.0) | 11 (22.9) |
| Immunohistochemical staining-No.(%) | | |
| CD117 positive | 4 (100.0) | 37 (77.1) |
| DOG-1 positive | 4 (100.0) | 42 (87.5) |
| CD34 positive | 1 (25.0) | 43 (89.6) |
| Risk classification-No.(%) | | |
| Very low to low risk | 0 (0.0) | 21 (43.8) |
| Medium risk | 0 (0.0) | 12 (25.0) |
| High risk | 4 (100.0) | 15 (31.2) |
| Mitotic accounts | | |
| ≤5/5 mm^2^ | 0 (0.0) | 43 (89.6) |
| 5-10/5 mm^2^ | 1 (25.0) | 4 (8.3) |
| >10/5 mm^2^ | 3 (75.0) | 1 (2.1) |
