# Supplementary material for: Anaplastic lymphoma kinase expression in PDGFRA-mutated gastrointestinal stromal tumors probably correlates with poor prognosis
Source: World J Surg Oncol. 2023 Apr 29;21:138. doi: 10.1186/s12957-023-03019-4 (PMC10148552; doi:10.1186/s12957-023-03019-4)
Supplement: Supplementary file 3 — Additional file 3: Supplementary Figure S1. Examination images of case 2. (A) CT revealed an irregular mass sized 18.5×7.3×28.2 cm located in the left abdominal cavity. The margins of the lesion could not be clearly discriminated from the stomach and intestinal walls (red arrow). (B) The tumor showed an epithelioid growth pattern by H&E staining (200×). (C) The tumor cells showed a positive cytoplasmic signal for CD117 (200×). (D) The tumor cells showed a positive cytoplasmic signal for DOG1 (200×). (E) The tumor cells showed a negative cytoplasmic signal for SMA (200×). (F) ALK IHC showed positive strong and diffuse staining by the 1A4 clone (200×). (G) ALK IHC showed positive strong and diffuse staining by the D5F3 clone (200×). (H) A break-apart fluorescent in situ hybridization (FISH) assay did not reveal ALK rearrangement (1000×). Supplementary Figure 2. Examination images of case 3. (A) Preoperative CT showed a mixed slightly low-density mass of approximately 15.7×7.6 cm in the right-middle abdomen. Contrast-enhanced scanning showed uneven enhancement, with multiple areas of unenhanced necrosis (red arrow). (B) The tumor demonstrated epithelioid and spindle growth by H&E staining (200×). (C) The tumor cells showed a positive cytoplasmic signal for CD117 (200×). (D) The tumor cells showed a positive cytoplasmic signal for DOG1 (200×). (E) The tumor cells showed a negative cytoplasmic signal for SMA (200×). (F) ALK IHC showed strong positive staining by the 1A4 clone (200×). (G) ALK IHC showed strong positive staining by the D5F3 clone (200×). (H) A break-apart fluorescent in situ hybridization (FISH) assay did not identify ALK rearrangement, but some tumor cells were identified as having a copy number >2 (1000×). Supplementary Figure 3. The examination images of case 4. (A) Examination CT showed uneven enhanced density shadows in the pelvic cavity, right rectus abdominus region and deep right abdominal wall, the maximum size of which was 11.0 cm×6.7 cm (red arr [file 12957_2023_3019_MOESM3_ESM.docx]

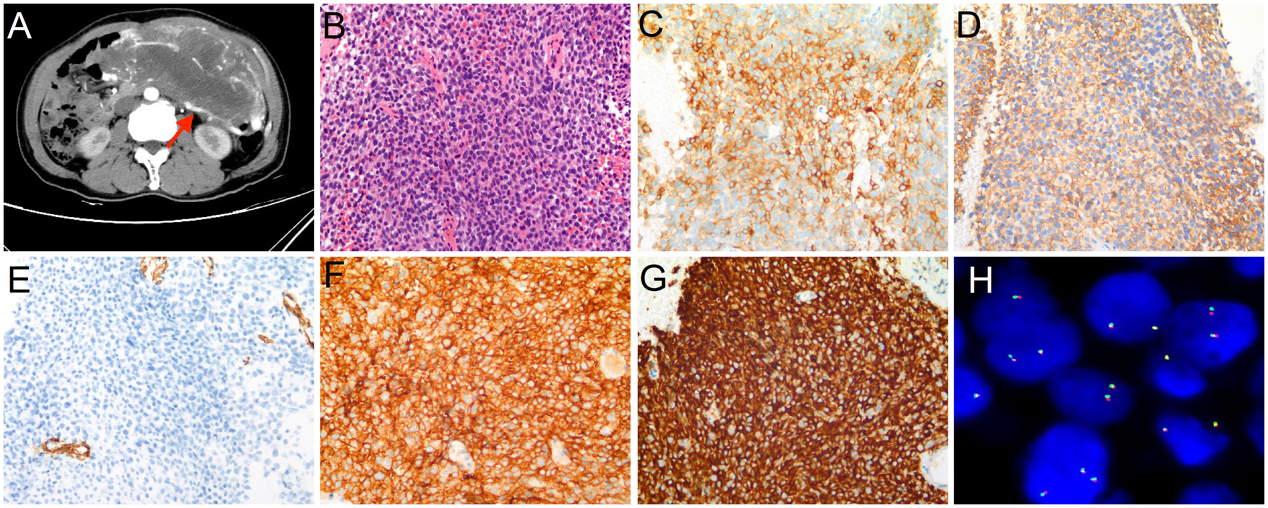


**Supplementary Figure 1.**Examination images of case 2. (A) CT revealed an irregular mass sized 18.5×7.3×28.2 cm located in the left abdominal cavity. The margins of the lesion could not be clearly discriminated from the stomach and intestinal walls (red arrow). (B) The tumor showed an epithelioid growth pattern by H&E staining (200×). (C) The tumor cells showed a positive cytoplasmic signal for CD117 (200×). (D) The tumor cells showed a positive cytoplasmic signal for DOG1 (200×). (E) The tumor cells showed a negative cytoplasmic signal for SMA (200×). (F) ALK IHC showed positive strong and diffuse staining by the 1A4 clone (200×). (G) ALK IHC showed positive strong and diffuse staining by the D5F3 clone (200×). (H) A break-apart fluorescent in situ hybridization (FISH) assay did not reveal *ALK* rearrangement (1000×).


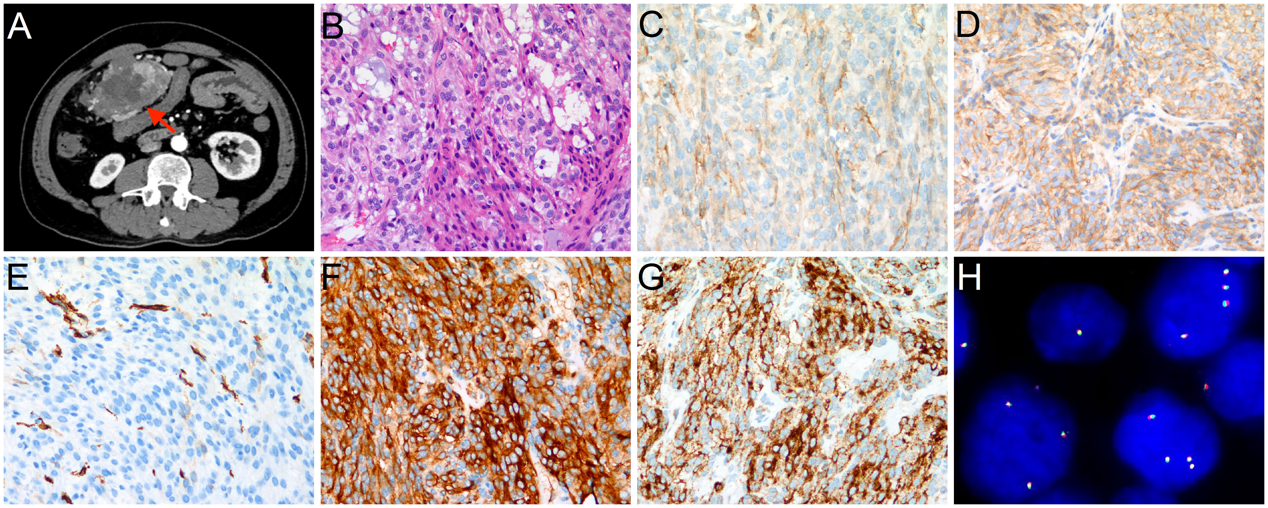


**Supplementary Figure 2.** Examination images of case 3. (A) Preoperative CT showed a mixed slightly low-density mass of approximately 15.7×7.6 cm in the right-middle abdomen. Contrast-enhanced scanning showed uneven enhancement, with multiple areas of unenhanced necrosis (red arrow). (B) The tumor demonstrated epithelioid and spindle growth by H&E staining (200×). (C) The tumor cells showed a positive cytoplasmic signal for CD117 (200×). (D) The tumor cells showed a positive cytoplasmic signal for DOG1 (200×). (E) The tumor cells showed a negative cytoplasmic signal for SMA (200×). (F) ALK IHC showed strong positive staining by the 1A4 clone (200×). (G) ALK IHC showed strong positive staining by the D5F3 clone (200×). (H) A break-apart fluorescent in situ hybridization (FISH) assay did not identify ALK rearrangement, but some tumor cells were identified as having a copy number >2 (1000×).


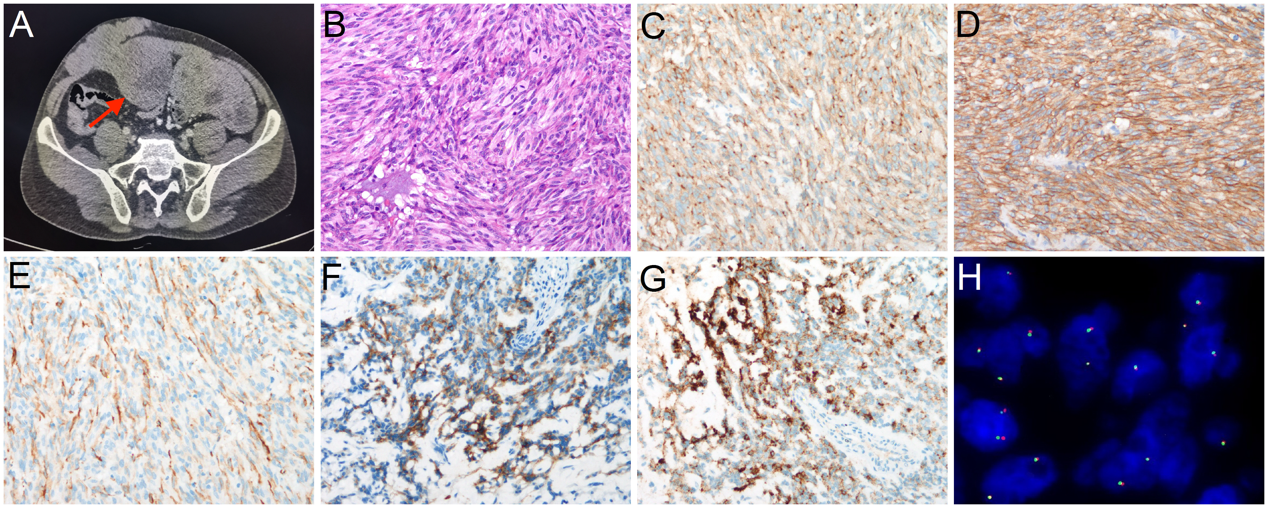


**Supplementary Figure 3.** The examination images of case 4. (A) Examination CT showed uneven enhanced density shadows in the pelvic cavity, right rectus abdominus region and deep right abdominal wall, the maximum size of which was 11.0 cm×6.7 cm (red arrow). (B) The tumor demonstrated a spindle growth pattern stained by H&E staining (200×). (C) The tumor cells showed a positive cytoplasmic signal for CD117 (200×). (D) The tumor cells showed a positive cytoplasmic signal for DOG1 (200×). (E) The tumor cells showed a positive cytoplasmic signal for SMA (200×). (F) ALK IHC showed positive staining by the 1A4 clone (200×). (G) ALK IHC showed strong positive staining by the D5F3 clone (200×). (H) A break-apart fluorescent in situ hybridization (FISH) assay did not reveal *ALK* rearrangement (1000×).


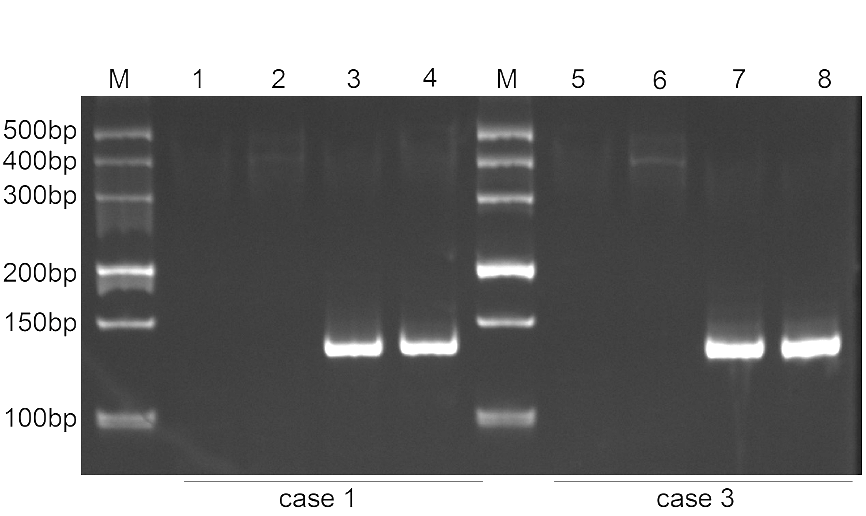
**Supplementary Figure 4.** PAGE electrophoresis results of RT-PCR products of two available pairwise tissues. Lane 1-4 and 5-8 show the electrophoresis results of case 1 and case 3. Lane M, DNA marker; Lane 1/5, PCR amplification product obtained with *ALK* gene primers from normal tissues; Lane 2/6, PCR amplification product obtained with *ALK* gene primers from tumor tissues; Lane 3/7, PCR amplification product obtained with *GAPDH* gene primers from normal tissues; Lane 4/8, PCR amplification product obtained with *GAPDH* gene primers from tumor tissues.
